# Genomic and transcriptomic correlates of deep prostate–specific antigen response in patients with metastatic androgen pathway modulation–sensitive prostate cancer

**DOI:** 10.1093/oncolo/oyag368

**Published:** 2026-09-17

**Authors:** Rana R McKay, Deepak Kilari, Binyam Yilma, Unnati Jariwala, Arya Ashok, Stamatina Fragkogianni, Jacob Mercer, Neeraj Agarwal, Emmanuel S Antonarakis

**Affiliations:** Moores Cancer Center, University of California San Diego, La Jolla, CA 92093, United States; Department of Medicine, Medical College of Wisconsin, Milwaukee, WI 53226, United States; Tempus AI, Inc, Chicago, IL 60654, United States; Tempus AI, Inc, Chicago, IL 60654, United States; Tempus AI, Inc, Chicago, IL 60654, United States; Tempus AI, Inc, Chicago, IL 60654, United States; Tempus AI, Inc, Chicago, IL 60654, United States; Department of Internal Medicine, University of Utah, Salt Lake City, UT 84112, United States; Department of Medicine, Masonic Cancer Center, University of Minnesota, Minneapolis, MN 55455, United States

**Keywords:** hormone sensitive, PSA response, PSA nadir, molecular correlates, prognosis, biomarkers

## Abstract

**Background:**

Despite advances in metastatic androgen pathway modulation-sensitive prostate cancer (mAPMS) treatment, outcomes remain heterogeneous. Achieving a post-treatment undetectable prostate-specific antigen (PSA) is a strong prognostic marker. We aimed to identify genomic and transcriptomic determinants of PSA response in a real-world clinical-genomic cohort.

**Patients and Methods:**

Patients with mAPMS who underwent DNA (Tempus xT) and, in a subset, RNA (Tempus xR) sequencing were identified from the Tempus Lens database. Inclusion required stage IV disease within 90 days of sample collection and obtained within 12 months before or 3 months after treatment initiation. Patients with PSA at 6 months (*n* = 525) were classified as PSA-Low (<0.1 ng/mL, *n* = 240) or PSA-High (≥0.1 ng/mL, *n* = 285). Overall survival (OS) was assessed by 6-month landmark analysis with delayed-entry adjustment. Logistic and Cox models were adjusted for clinical variables. Sensitivity analyses used a relative definition of >95% PSA decline from baseline.

**Results:**

Baseline PSA was lower in PSA-Low versus PSA-High patients (24 vs 36 ng/mL, *P* = .01). *SPOP* (17% vs 11%) and *ZFHX3* (2.5% vs 6%) alterations differed between groups, but neither persisted after adjustment. Using the relative definition, *ZMYM3* and *JAK1* alterations were independently associated with failure to achieve a deep PSA response. Expression of *PSMA*, *TROP2*, *B7-H3*, and *STEAP1* did not differ between groups. PSA-Low status was independently associated with improved OS, as was deep relative response.

**Conclusion:**

Deep PSA response at 6 months correlates with improved OS in mAPMS. Integrating molecular markers with PSA response may inform treatment intensification or de-escalation strategies.

Implications for practice This real-world clinical-genomic analysis examined molecular correlates of deep PSA response in metastatic androgen pathway modulation-sensitive prostate cancer. Deep PSA response at 6 months was independently associated with improved survival, whether defined by an absolute or relative threshold. *SPOP* enrichment among deep responders did not persist after adjustment for baseline disease characteristics, whereas *ZMYM3* and *JAK1* alterations were independently associated with failure to achieve a deep response. Baseline genomic features may help refine prognostic assessment in mAPMS but require validation before clinical application.

## Introduction

The treatment landscape for metastatic androgen pathway modulation-sensitive prostate cancer (mAPMS, previously termed metastatic hormone-sensitive prostate cancer) has evolved substantially over the past decade from androgen deprivation therapy (ADT) alone to treatment intensification strategies combining ADT with androgen receptor pathway inhibitors (ARPIs), with or without docetaxel chemotherapy.^1–8^ Multiple phase 3 randomized trials have demonstrated significant improvements in overall survival (OS) with intensified treatment approaches, establishing these combinations as the current standard of care.^1–7^ Despite these advances, treatment responses remain heterogeneous, and identifying patients most likely to derive benefit from specific strategies remains an ongoing challenge.

Early on-treatment markers that predict long-term outcomes are increasingly being explored to guide clinical decision-making. Prostate-specific antigen (PSA) response has emerged as a clinically meaningful marker of treatment efficacy. Specifically, achieving an undetectable or deep PSA response (typically defined as <0.2 ng/mL) at 6 months after treatment has been associated with improved OS (Table 1).^9–18^

**Table 1.** Summary of 6-month PSA response data from phase 3 trials in mAPMS.

Trial	Treatment arms	PSA <0.2 ng/mL rate at 6 months (experimental)	PSA <0.2 ng/mL rate at 6 months (control)	**HR for OS (undetectable vs detectable PSA)** ^a^	Reference
**ARASENS**	Darolutamide + ADT + Docetaxel vs Placebo + ADT + Docetaxel	43% HV; 67% LV	22% HV; 32% LV	HR 0.49 (95% CI, 0.37-0.65) at 24 weeks	Saad et al.^11^
**PEACE-1**	Abiraterone + Prednisone + ADT + Docetaxel vs Placebo + ADT + Docetaxel	51%^b^	25%^b^	OS: NE vs 3.5 years (*P* = .0007)^b^	Gravis Mescam et al.^16^
**TITAN**	Apalutamide + ADT vs Placebo + ADT	61%	24%	HR 0.66 (95% CI, 0.44-1.00)	Roy et al.^12^
**ARCHES**	Enzalutamide + ADT vs Placebo + ADT	68%^c^	18%^c^	HR 0.24 (95% CI, 0.17-0.34)	Armstrong et al.^18^
**ENZAMET**	Enzalutamide + ADT vs NSAA + ADT (concurrent docetaxel permitted)	67%^d^	48%^d^	8-year OS: 55% vs 23% (PSA ≤0.2 vs >0.2, *P* < .001)	Zhang et al.^8^
**ARANOTE**	Darolutamide + ADT vs Placebo + ADT	55% HV; 83% L^e^	16% HV; 25% L^e^	HR 0.14 (95% CI, 0.09-0.21)^f^	Saad et al.^11^
**LATITUDE**	Abiraterone + Prednisone + ADT vs Placebo + ADT	58%^b^	13%^b^	HR 0.44 (95% CI, NR)	Matsubara et al.^15^
**CHAARTED**	ADT + Docetaxel vs ADT	32%	19%	HR 0.41	Tripathi et al.^9^
**SWOG S1216**	ADT + Orteronel vs ADT + Bicalutamide	61%^d^	46%^d^	HR 0.20	Parikh et al.^13^

Abbreviations: ADT, androgen deprivation therapy; HR, hazard ratio; HV, high-volume disease; LV, low-volume disease; mAPMS, androgen pathway modulation–sensitive prostate cancer; NE, not estimable; NR, not reported; NSAA, non-steroidal anti-androgen; PSA, prostate-specific antigen.

a HR reported for experimental arm comparing patients achieving undetectable PSA vs those with detectable PSA.

b Rate reported at 8 months.

c Rate reported at any time during treatment.

d Rate reported at 7 months.

e Rate reported at 24 weeks by disease volume.

f HR reflects radiographic progression-free survival, not overall survival.

Advances in tumor molecular profiling have identified prognostic genomic alterations in prostate cancer.^19–21^ Alterations in tumor suppressor genes such as *TP53*, *RB1*, and *PTEN* are associated with aggressive disease biology and inferior outcomes. Similarly, deleterious alterations in *BRCA1/2* and other DNA damage repair genes may also portend a worse prognosis with standard systemic therapies. Conversely, *SPOP* alterations have been associated with favorable outcomes in patients with advanced prostate cancer.^22–24^ Similarly, *FOXA1* alterations also portend more favorable outcomes.^1^^,^^25^ Despite these advances in understanding disease biology, the correlation between baseline molecular features and achievement of deep PSA response to first-line systemic therapy has not been well characterized.

Identifying molecular predictors of deep PSA response could have important clinical implications, potentially informing treatment intensification strategies for patients unlikely to achieve optimal responses or guiding de-escalation approaches for those with favorable molecular profiles. The clinical relevance of depth of PSA response is underscored by prospective trials now designed explicitly around this metric: TRIPLE-SWITCH (SWOG/CCTG-PR26; NCT06592924) is evaluating the addition of docetaxel to ARPIs in patients with mAPMS and suboptimal PSA response, while A-DREAM (Alliance A032101; NCT05241860) is testing ADT interruption in patients achieving exceptional responses to ARPI, together illustrating the emerging paradigm of response-adaptive therapy in mAPMS. We hypothesized that baseline molecular features would differ between patients who achieve deep PSA responses compared to those with suboptimal responses. We leveraged a large real-world clinical-genomic cohort to identify molecular determinants associated with achieving a favorable PSA response at 6 months in patients with mAPMS treated with ADT-based combination therapy. We additionally explored gene expression of emerging therapeutic targets to determine whether differences exist based on depth of PSA response.

## Methods

### Study cohort and sample selection

The Tempus Lens Platform (Tempus AI, Inc., Chicago, IL), a de-identified real-world clinico-genomic database, was queried to identify patients with a primary diagnosis of prostate cancer and filtered to those who underwent next-generation sequencing with the Tempus xT (DNA, 648 gene panel) assay, with a subset additionally having the Tempus xR (RNA, whole transcriptome) assay. Patients were further restricted to those with stage IV disease documented within 90 days of sample collection. To identify patients with mAPMS, we selected patients who had samples collected prior to treatment initiation or within 3 months of initiating systemic therapy. Eligible first-line ADT-based regimens included ADT alone, ADT plus docetaxel, ADT plus an ARPI (abiraterone, enzalutamide, apalutamide, or darolutamide), or ADT plus docetaxel plus ARPI. Samples collected more than 3 months after ADT initiation were categorized as castration-resistant and excluded from analysis. For patients with multiple sequenced samples, eligible biopsies were restricted to those collected within 1 year prior to or up to 15 days after first-line treatment initiation, with the sample collected closest to treatment initiation selected for analysis. Additionally, we required PSA data at 6 months (±45 days) resulting in a cohort of 525 patients (Figure S1, see online supplementary material for a color version of this figure). All analyses were performed using de-identified data obtained from the Tempus Lens database under an exempt determination issued by the Advarra, Inc. Institutional Review Board. As the study involved only de-identified retrospective data, informed consent was waived. The study was conducted in accordance with the ethical principles of the Declaration of Helsinki.

### PSA response group definitions

Patients were categorized into 2 groups based on depth of PSA response at 6 months. PSA-Low was defined as mAPMS patients achieving a PSA level of <0.1 ng/mL at 6 months (±45 days) post-treatment initiation. PSA-High was defined as mAPMS patients with a PSA level of ≥0.1 ng/mL at 6 months (±45 days) post-treatment initiation. This threshold was selected to provide a discriminatory cutoff for identifying patients with deep PSA responses. Given that an absolute threshold does not account for heterogeneity in baseline PSA, a sensitivity analysis was performed using a relative definition of deep response, defined as a >95% decline from baseline PSA at 6 months (Deep Response, *n* = 366; No Deep Response, *n* = 55).

### Genomic analysis

All tumor samples underwent DNA next-generation sequencing. Genomic alterations, including pathogenic/likely pathogenic single nucleotide variants, insertions/deletions, and copy number variations were aggregated at the gene level. Genes were included in the genomic landscape analysis if alterations were detected in at least 1% of patients. Tumor mutation burden (TMB) was calculated by dividing the number of nonsynonymous mutations by the megabase size of the panel. All non-silent genomic coding mutations, including missense, indel, and stop-loss variants with coverage greater than 100 × and an allelic fraction greater than 5%, were counted as non-synonymous mutations. Microsatellite instability (MSI) status was determined by previously published methods.^26–28^

### Transcriptomic analysis

RNA sequencing data were normalized by computing transcripts per million (TPM) for all protein-coding transcripts and represented as log2(TPM). Expression levels of therapeutic targets, including *PSMA* (*FOLH1*), *STEAP1*, *STEAP2*, *DLL3*, *CEACAM5*, *TROP2* (*TACSTD2*), *NECTIN4*, *B7-H3* (*CD276*), and B7-H4 (*VTCN1*), were analyzed for their association with PSA response groups. Androgen receptor (*AR*) and neuroendocrine prostate cancer (NEPC) signaling activity were evaluated using established gene expression signatures and compared between PSA-High and PSA-Low groups. Signature scores were calculated as the mean expression of genes within each signature set. Immune cell infiltration was estimated using the quanTIseq algorithm, which deconvolutes RNA sequencing-based gene expression data via a support vector regression model to estimate proportions of immune cell populations within the tumor microenvironment, including B cells, M1 macrophages, M2 macrophages, monocytes, neutrophils, natural killer (NK) cells, CD4+ T cells, CD8+ T cells, regulatory T cells, and dendritic cells.^29^

### Differential gene expression

Transcriptomic differences at the gene level between the PSA-High and PSA-Low groups were assessed via whole-transcriptome differential gene expression (DGE) analysis among patients with available RNA sequencing data. Statistical comparisons were performed using a covariate-adjusted Wilcoxon rank-sum test, controlling for age at biopsy and tumor purity to mitigate confounding effects.

### Single-sample gene set enrichment analysis

To quantify pathway-specific transcriptional activity, single-sample Gene Set Enrichment Analysis (ssGSEA) was performed using the GSEABase package in R. This method computes an enrichment score for each individual sample based on the relative expression ranking of genes within predefined gene sets, including AR and NEPC. Median enrichment scores were then compared between the PSA-High and PSA-Low groups using a Wilcoxon rank-sum test.

### Statistical analysis

All clinical and molecular characteristics were compared between patients in the PSA-Low versus PSA-High groups. Categorical variables were reported as frequencies and percentages, with *P*-values calculated using χ² and Fisher’s exact tests as appropriate. Continuous variables were reported as medians with interquartile ranges, with *P*-values calculated using Wilcoxon rank-sum tests. PSA strata, PSA ≥0.1 ng/mL ng/mL (PSA-High) and PSA <0.1 ng/mL (PSA-Low), and the landmark timepoint was defined at 6 months after ADT start.

To assess whether genomic features were independently associated with depth of PSA response, per-gene multivariable logistic regression models were fit with PSA response group as the outcome and each genomic alteration as the exposure. Associations were expressed as odds ratios (OR) with 95% confidence intervals (CI), representing the odds of alteration in the deep response group relative to the comparator group, with an OR greater than 1 indicating enrichment among deep responders. Models were adjusted for baseline PSA, bone metastases, and first-line treatment category, selected because these were the baseline characteristics that differed between PSA response groups and are established determinants of PSA kinetics. The covariate set was kept deliberately parsimonious given the number of alteration-positive patients. Genes for which estimates were unstable, owing to sparse alteration-by-outcome cells (minimum cell count <5) or complete separation, were reported as non-estimable rather than as unreliable point estimates. Analyses were repeated using the relative >95% PSA decline definition. Genomic analyses were exploratory and were not corrected for multiple comparisons.

OS was evaluated using a 6-month landmark analysis, defined as 6-month post-initiation of first-line treatment to death or last known follow-up. Median OS from this landmark was estimated using Kaplan-Meier methods, with differences between PSA response groups assessed via log-rank tests. Median follow-up was estimated using the reverse Kaplan-Meier method. Hazard ratios (HR) and 95% CI were estimated to be using Cox proportional hazards regression models, adjusting for age at biopsy, baseline PSA, first-line treatment category, Gleason score, and metastatic sites (bone, lymph node, visceral). All sequenced samples were obtained at or prior to first-line treatment initiation, but sequencing was in some cases performed later. Because cohort inclusion required an available sequencing result, patients could not be observed before that result existed. To mitigate the resulting immortal time bias, a delayed-entry (left-truncated) adjustment was applied, with patients entering the risk set at the later of their sequencing date or the 6-month landmark. Patients sequenced after the landmark therefore enter at later timepoints, which produces an expected transient increase in the number at risk early in follow-up. Mortality was ascertained from electronic health records augmented by tokenized third-party mortality data, and event-free patients were censored at last curated follow-up. Statistical significance was set at *P* ≤ .05 for all 2-sided tests. Missing observations were excluded on an analysis-by-analysis basis. All analyses were performed using R programming language ([R version 4.5.0 (2025-04-11]).

## Results

### Patient characteristics

A total of 525 patients with mAPMS met inclusion criteria and were included in the final analysis (Figure S1, see online supplementary material for a color version of this figure). Patients were stratified by 6-month post-treatment PSA response into PSA-High (≥0.1 ng/mL; *n* = 285) and PSA-Low (<0.1 ng/mL; *n* = 240) groups. Baseline characteristics were generally well balanced between groups (Table 2). Median age at diagnosis was 68 years in both groups. The majority of patients were white (58% PSA-High, 52% PSA-Low), and most had metastatic diagnosis at first inclusion (92% PSA-High, 89% PSA-Low). Patients in the PSA-High group had a significantly higher prevalence of bone metastases compared to the PSA-Low group (56% versus 42%, *P* < .001). The distribution of first-line systemic therapy was similar between groups, with ADT plus ARPI being the most common regimen (64% PSA-High, 65% PSA-Low). At treatment initiation, median baseline PSA was numerically higher in the post-treatment PSA-High group compared to the PSA-Low group (36 ng/mL versus 24 ng/mL), with median post-treatment PSA at 6 months at 0.14 ng/mL in the PSA-High group and 0.04 ng/mL in the PSA-Low group.

**Table 2.** Baseline patients and disease characteristics by PSA response group.

Characteristic	PSA-high Median (Q1, Q3) or *N* (%) (*n* = 285)	PSA-low Median (Q1, Q3) or *N* (%) (*N* = 240)	*P*-value
**Age at diagnosis**	67 (61, 73)	68 (61, 73)	.8
**Race**			.5
** White**	165 (58)	135 (52)	
** Asian**	39 (14)	38 (16)	
** Black/African American**	6 (2.1)	4 (1.7)	
** Other race**	23 (8.1)	16 (6.7)	
** Unknown**	52	57	
**Ethnicity**			**.003**
** Not Hispanic**	187 (66)	126 (53)	
** Hispanic**	16 (5.6)	11 (4.6)	
** Unknown**	82	103	
**Time from documented primary diagnosis to initial metastatic diagnosis**			.5
** Within 3 months**	245 (92)	201 (89)	
** 3-6 months**	3 (1.1)	3 (1.3)	
** More than 6 months**	19 (7.1)	22 (9.7)	
** Unknown**	18	14	
**Sites of metastatic disease**			
** Bone**	161 (56)	100 (42)	**<.001**
** Lymph node**	100 (35)	81 (34)	.8
** Lung**	12 (4.2)	9 (3.8)	.8
** Liver**	6 (2.1)	2 (0.8)	.3
**Baseline PSA, ng/mL^a^**	36 (10, 139)	24 (4, 91)	**.012**
**Time from metastatic diagnosis to first-line systemic therapy for mAPMS, months**	0.85 (0.26, 1.58)	0.97 (0.39, 1.78)	.068
**Time from sample collection to first-line systemic therapy for mAPMS, months**	−0.69 (−1.38, −0.20)	−0.85 (−1.69, −0.30)	**.046**
**First-line systemic therapy for mAPMS**			.3
** ADT monotherapy**	50 (18)	37 (15)	
** ADT + ARPI**	181 (64)	155 (65)	
** ADT + ARPI + docetaxel**	44 (15)	45 (19)	
** ADT + docetaxel**	10 (3.5)	3 (1.3)	

PSA-low defined as PSA <0.1 ng/mL at 6 months; PSA-high defined as PSA ≥0.1 ng/mL at 6 months. *P*-values were calculated using Wilcoxon rank-sum test for continuous variables and Fisher’s exact test or Pearson’s chi-squared test for categorical variables. Bolded values are statistically significant.

Abbreviations: ADT, androgen deprivation therapy; ARPI, androgen receptor pathway inhibitor; mAPMS, metastatic androgen pathway modulation–sensitive prostate cancer; PSA, prostate-specific antigen; Q1, first quartile; Q3, third quartile.

a Within 45 days of first-line treatment start.

In the sensitivity cohort defined by relative PSA decline, 366 patients achieved a deep response (>95% decline from baseline) and 55 did not (Table S1). Patients without a deep relative response had markedly lower baseline PSA (median 1 versus 42 ng/mL, *P* < .001), fewer bone metastases (49% versus 68%, *P* = .005), and a longer interval from metastatic diagnosis to first-line therapy (1.15 versus 0.72 months, *P* = .002). Age and the distribution of first-line systemic therapy were comparable between groups.

### Genomic alterations

Genomic alterations were included if present in ≥1% of patients (Figure 1, Figures S1 and S2 [see online supplementary material for a color version of these figures], Table S2). In the unadjusted analyses, *SPOP* mutations were significantly enriched in the post-treatment PSA-Low group compared to the PSA-High group (17% versus 11%, OR 1.70, 95% CI, 1.02-2.83, *P* = .039). Conversely, *ZFHX3* alterations showed a trend toward enrichment in the PSA-High group (6.0% versus 2.5%, *P* = .053). No significant between-group differences were observed in any other commonly altered genes, including *TP53* (31% versus 30%, *P* = .7), *PTEN* (25% versus 20%, *P* = .2), *RB1* (3.2% versus 6.2%, *P* = .091), *FOXA1* (14% versus 10%, *P* = .2), or *BRCA2* (6.3% versus 3.8%, *P* = .2). After adjustment for clinical variables, the *SPOP* association was attenuated and no longer statistically significant (adjusted OR 1.41, 95% CI, 0.79-2.50, *P* = .24), as was the *ZFHX3* association (adjusted OR 0.54, 95% CI, 0.18-1.57, *P* = .26). No genomic alteration reached statistical significance in the adjusted model. The strongest residual signals were *RB1* (adjusted OR 2.44, 95% CI, 0.88-6.76, *P* = .086) and *APC* (adjusted OR 1.66, 95% CI, 0.91-3.02, *P* = .096), both directionally enriched among PSA-Low patients.

**Figure 1.**
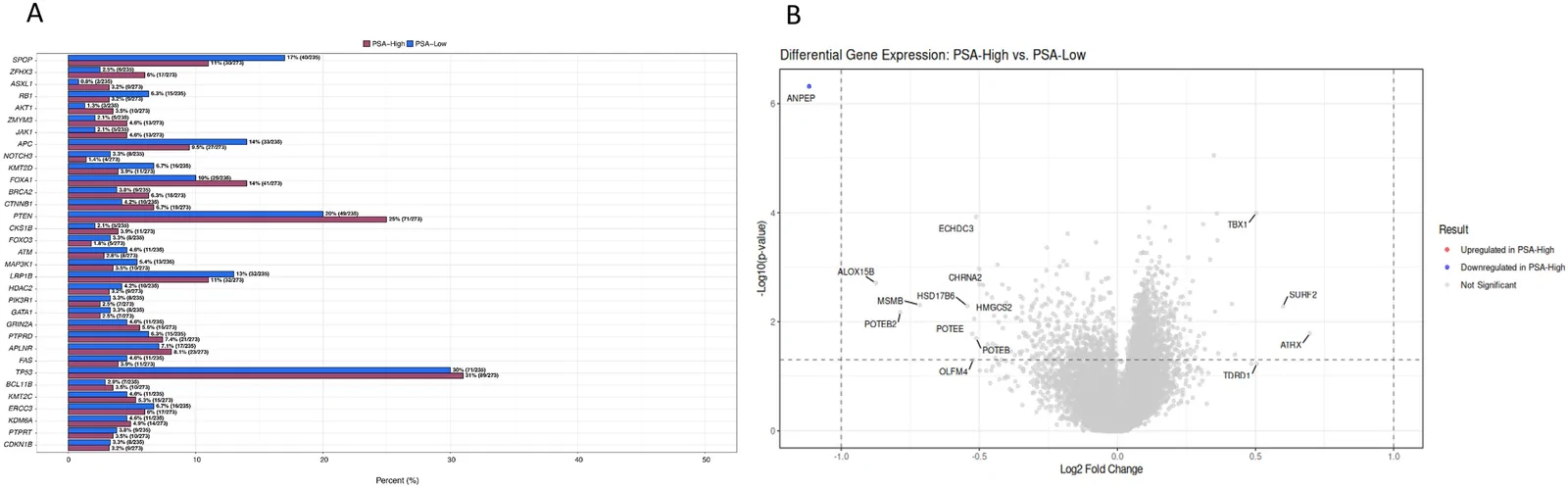
(A) Bar graph of mutation frequencies by PSA response groups. (B) Volcano plot of differentially expressed genes. PSA-low was defined as PSA <0.1 ng/mL and PSA-high as PSA ≥0.1 ng/mL at 6 months after initiation of first-line systemic therapy. Abbreviations: PSA, prostate-specific antigen.

In a sensitivity analysis using a relative definition of deep response, a distinct set of alterations was enriched among patients who did not achieve a deep response (Table S3). In unadjusted analyses, these included *JAK1* (13% versus 2.5%; OR 0.17, 95% CI, 0.06-0.49, *P* = .002), *ZMYM3* (9.1% versus 3.0%; OR 0.31, 95% CI, 0.10-0.93, *P* = .045), and *TP53* (44% versus 30%; OR 0.54, 95% CI, 0.30-0.96, *P* = .035). After adjustment for the same clinical covariates, *ZMYM3* (adjusted OR 0.10, 95% CI, 0.02-0.59, *P* < .05) and *JAK1* (adjusted OR 0.24, 95% CI, 0.06-0.94, *P* < .05) remained independently associated with failure to achieve a deep relative response, whereas the *TP53* association did not persist (adjusted OR 0.63, 95% CI, 0.31-1.26, *P* = .19). *SPOP* was not enriched among deep relative responders in either unadjusted or adjusted analyses (adjusted OR 0.47, 95% CI, 0.13-1.67, *P* = .24).

TMB was similar between groups, with the majority of patients classified as TMB-low (<10 mutations/Mb; 97% in both groups). MSI-high status was rare and comparable between groups (2.5% PSA-High versus 2.1% PSA-Low, *P* = .8).

### Gene expression analysis

RNA sequencing data were available for 418 patients (*n* = 224 PSA-High, *n* = 194 PSA-Low). Expression of therapeutic target genes did not differ significantly between PSA response groups (Table 3). *FOLH1* (PSMA), *STEAP1*, *STEAP2*, *CD276* (B7-H3), *TACSTD2* (TROP2), and *KLK2* showed comparable expression levels regardless of PSA response. Among the top differentially expressed genes, several were expressed at higher levels in the PSA-Low group, including *ANPEP* (median 6.36 versus 4.94, *P* < .001), *ECHDC3* (*P* < .001), *CHRNA2* (*P* = .002), *ALOX15B* (*P* = .002), *HMGCS2* (*P* = .002), *HSD17B6* (*P* = .004), *MSMB* (*P* = .006), *POTEB2* (*P* = .012), *POTEE* (*P* = .024), *OLFM4* (*P* = .035), and *POTEB* (*P* = .046). Conversely, *TBX1* (median 6.03 versus 5.65, *P* < .001), *SURF2* (median 4.73 versus 1.43, *P* = .003), and *ATRX* (median 7.14 versus 6.76, *P* = .009) were expressed at higher levels in the PSA-High group. The ssGSEA signature scores for *AR* and NEPC did not differ between the PSA-High and PSA-Low groups (median 0.62 versus 0.62, *P* = .6; and median 0.26 versus 0.27, *P* = .9, respectively).

**Table 3.** RNA gene expression by PSA response group.

Genes	PSA-high Median (Q1, Q3) TPM	PSA-low Median (Q1, Q3) TPM	*P*-value
**Therapeutic target genes**	*N* = 224	*N* = 194	
** *CD276 (B7-H3)***	5.98 (5.61, 6.49)	5.95 (5.48. 6.38)	.14
** *FOLH1 (PSMA)***	10.29 (9.40, 10.90)	10.13 (9.13, 10.81)	.2
** *CEACAM5***	0.64 (0.42, 1.33)	0.67 (0.47, 1.46)	.2
** *DLL3***	0.42 (0.16, 0.83)	0.38 (0.13, 0.70)	.3
** *NECTIN4***	4.07 (3.63, 4.48)	4.15 (3.71, 4.48)	.5
** *STEAP2***	9.66 (9.07, 10.06)	9.63 (9.13, 10.11)	.7
** *VTCN1 (B7-H4)***	0.77 (0.27, 1.42)	0.80 (0.26, 1.52)	.7
** *STEAP1***	9.58 (9.05, 9.98)	9.51 (9.05, 10.03)	.7
** *TACSTD2 (TROP2)***	7.62 (7.11, 8.06)	7.61 (7.12, 8.04)	.9
** *KLK2***	11.20 (10.63, 11.52)	11.19 (10.49, 11.55)	>.9
**Differentially expressed genes**	*N* = 224	*N* = 194	
** *ANPEP***	4.94 (3.76, 6.74)	6.36 (4.48, 8.67)	<.001
** *TBX1***	6.03 (5.31, 6.51)	5.65 (3.98, 6.31)	<.001
** *ECHDC3***	4.12 (2.58, 4.88)	4.56 (3.66, 5.12)	<.001
** *CHRNA2***	4.35 (2.91, 5.36)	4.84 (3.68, 5.66)	.002
** *ALOX15B***	3.30 (1.60, 6.65)	5.05 (1.96, 7.54)	.002
** *HMGCS2***	3.64 (2.50, 5.23)	4.38 (3.18, 5.74)	.002
** *SURF2***	4.73 (1.21, 5.42)	1.43 (0.90, 5.19)	.003
** *HSD17B6***	4.99 (3.91, 5.75)	5.30 (4.38, 6.42)	.004
** *MSMB***	7.44 (5.62, 9.25)	8.30 (6.49, 9.48)	.006
** *ATRX***	7.14 (1.59, 7.48)	6.76 (1.48, 7.33)	.009
** *POTEB2***	1.50 (0.34, 4.27)	2.74 (0.51, 5.96)	.012
** *POTEE***	7.51 (5.47, 9.34)	8.08 (6.67, 9.45)	.024
** *OLFM4***	2.46 (1.05, 4.14)	2.88 (1.17, 5.19)	.035
** *POTEB***	4.01 (2.02, 5.80)	4.64 (2.77, 6.29)	.046
** *TDRD1***	5.89 (2.00, 7.18)	5.02 (1.66, 6.85)	.056

PSA-low defined as PSA <0.1 ng/mL at 6 months; PSA-high defined as PSA ≥0.1 ng/mL at 6 months. Values represent log₂-transformed transcripts per million (TPM). Data presented as median (Q1, Q3). *P*-values were calculated using Wilcoxon rank-sum test.

Abbreviations: PSA, prostate-specific antigen; Q1, first quartile; Q3, third quartile; TPM, transcripts per million.

### Tumor immune microenvironment

Immune cell infiltration was estimated using the quanTIseq algorithm in 391 patients (*n* = 214 PSA-High, *n* = 177 PSA-Low) (Table S4). No significant differences in immune cell populations were observed between post-treatment PSA response groups. M2 macrophages and neutrophils were the most abundant immune cell types in both groups. CD8+ T cells, CD4+ T cells, regulatory T cells, NK cells, B cells, M1 macrophages, dendritic cells, and monocytes showed comparable infiltration levels between groups (all *P* > .2).

### Overall survival

Among 405 patients with available survival data (*n* = 226 PSA-High, *n* = 179 PSA-Low), 75 deaths were observed (*n* = 53 PSA-High, *n* = 22 PSA-Low) (Figure 2). Because a delayed-entry risk-set adjustment was applied, the number-at-risk table in Figure 2 begins with the subset of patients who had both survived to the 6-month landmark and been sequenced by that time (*n* = 87 PSA-High, *n* = 79 PSA-Low) and increases transiently thereafter as additional patients enter the risk set. Patients in the PSA-Low group had significantly longer OS compared to the PSA-High group (HR 0.54, 95% CI, 0.32-0.90, *P* = .017). Median follow-up time was comparable between the 2 cohorts, at 20 (95% CI, 17-23) months for the PSA-High group and 21 (95% CI, 16-23) months for the PSA-Low group. Median OS was 47 months (95% CI, 38-not reached) in the PSA-High group and was not reached (95% CI, 54-not reached) in the PSA-Low group. The median OS estimate in the PSA-High group was reached late in follow-up, when relatively few patients remained at risk, and should be interpreted with caution. Landmark survival was higher in the PSA-Low group at 24 and 36 months: 86% (95% CI, 78-92) versus 83% (95% CI, 75-89) at 24 months, and 83% (95% CI, 74-89) versus 66% (95% CI, 56-74) at 36 months. At 12 months, survival was 93% (95% CI, 87-96) in the PSA-Low group and 97% (95% CI, 92-99) in the PSA-High group. On multivariable analysis, PSA-Low remained independently associated with improved OS (adjusted HR 0.42, 95% CI, 0.21-0.84, *P* = .01).

**Figure 2.**
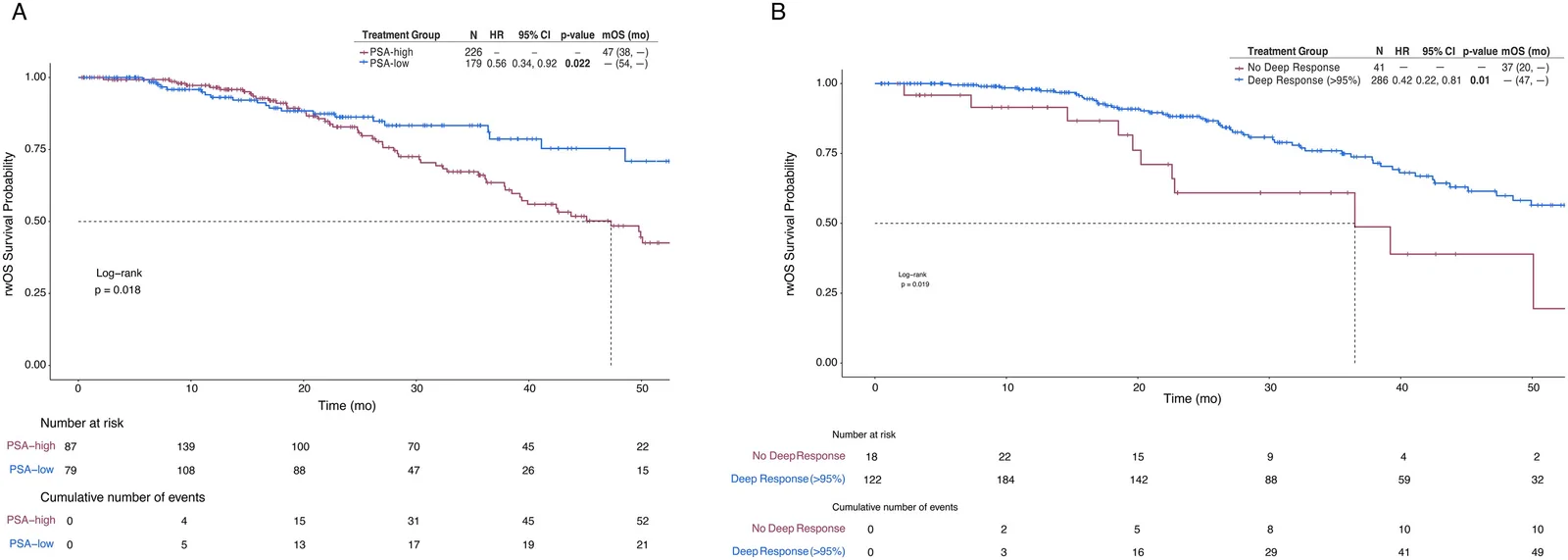
Kaplan-Meier curves comparing OS by depth of PSA response. (A) PSA-low (<0.1 ng/mL at 6 months) vs PSA-high (≥0.1 ng/mL at 6 months). (B) Deep response (>95% decline from baseline PSA at 6 months) vs no deep response. The dashed line indicates median OS. Abbreviations: HR, hazard ratio; mo, months; mOS, median overall survival; PSA, prostate-specific antigen.

A consistent survival advantage was observed when depth of response was defined by relative PSA decline (Figure 2). Among 327 patients with evaluable survival data in this sensitivity cohort (*n* = 286 deep response, *n* = 41 no deep response), 61 deaths occurred (*n* = 50 and *n* = 11, respectively). Median follow-up was 22 (95% CI, 19-25) months in the deep response group and 15 (4.3, 23) months in the no deep response group. Median OS was not reached (95% CI, 47-not reached) among patients achieving a >95% relative decline, compared with 37 months (95% CI, 20-not reached) among those who did not. Landmark survival was higher in the deep response group at every timepoint: 98% (95% CI, 95-99) versus 91% (95% CI, 70-98) at 12 months, 88% (95% CI, 82-92) versus 61% (95% CI, 37-78) at 24 months, and 75% (95% CI, 66-82) versus 61% (95% CI, 37-78) at 36 months. Achieving a deep relative response was associated with improved OS (HR 0.42, 95% CI, 0.22-0.81, *P* = .010), and this association persisted after adjustment for clinical variables (*n* = 267, 47 events; adjusted HR 0.43, 95% CI, 0.20-0.93, *P* = .031).

## Discussion

The present study leverages a large, real-world clinicogenomic database to characterize molecular features associated with PSA response in patients with mAPMS receiving contemporary first-line systemic therapy. Our principal finding is that depth of PSA response at 6 months is independently associated with OS, and that this association holds whether depth of response is defined by an absolute threshold (<0.1 ng/mL) or by relative decline from baseline (>95%), with comparable magnitude after adjustment for baseline disease characteristics. The two definitions identified different molecular correlates. Several associations observed with the absolute threshold, including enrichment of *SPOP* alterations among deep responders, were confounded by baseline disease characteristics and did not persist after adjustment. Using the relative definition, alterations in *ZMYM3* and *JAK1* remained independently associated with failure to achieve a deep response. DGE was observed between response groups, with PSA-Low patients demonstrating higher expression of genes associated with luminal differentiation and androgen-regulated transcriptional programs, whereas expression of therapeutic targets, immune cell infiltration, and AR/NEPC signaling did not differ between PSA groups. Together, these results begin to provide insights into the biological underpinnings of treatment response in mAPMS while underscoring that apparent genomic associations with PSA response may substantially reflect differences in baseline disease presentation.

*SPOP* mutations were enriched in patients achieving a deep PSA response in unadjusted analyses, consistent with prior studies demonstrating favorable outcomes in *SPOP*-mutant prostate cancer.^22–24^  *SPOP* encodes an E3 ubiquitin ligase adaptor protein that regulates AR degradation, and loss-of-function mutations increase AR protein stability, defining a subtype characterized by high AR activity and improved responses to ADT and ARPI.^19–21^ In our cohort, however, this enrichment did not persist after adjustment for clinical variables and was not enriched among deep responders by percent PSA decline. A similar pattern was observed for *ZFHX3*, a transcription factor implicated in AR signaling whose alterations have been associated with adverse outcomes.^30–32^ The *ZFHX3* enrichment among PSA-High patients likewise did not persist after adjustment for clinical variables.

The alterations that remained independently associated with depth of response were identified using the relative response definition. *ZMYM3* and *JAK1* alterations were each associated with failure to achieve a >95% PSA decline after adjustment for baseline characteristics. *ZMYM3* encodes a chromatin-associated zinc finger protein that functions within transcriptional corepressor complexes, and its loss may alter transcriptional programs downstream of AR signaling. *JAK1* is a central mediator of JAK/STAT signaling, a pathway implicated in lineage plasticity and the emergence of AR-independent phenotypes, offering a plausible mechanism by which *JAK1* alteration could blunt the depth of response to AR-directed therapy. *TP53* alterations were more frequent among patients without a deep relative response in unadjusted analysis, but were not independently associated after adjustment, and neither *PTEN*, *RB1*, *FOXA1*, nor *BRCA2* was independently associated under either definition.

RNA expression of established therapeutic targets, including *PSMA*, *STEAP1*, *STEAP2*, *B7-H3*, and *TROP2* did not differ between PSA response groups, suggesting that target RNA expression alone may not predict treatment response to existing therapies in mAPMS. Among the top differentially expressed genes, *ANPEP* showed the most significant association with deep PSA response. *ANPEP* encodes aminopeptidase N (CD13), a zinc-dependent metalloprotease involved in peptide processing and angiogenesis.^33^^,^^34^ Prior studies have demonstrated that ANPEP is downregulated in prostate cancer and that loss of expression is an adverse prognostic factor, suggesting that higher *ANPEP* expression in PSA-Low patients may reflect a more differentiated, treatment-responsive phenotype. Additional genes with significantly higher expression in PSA-Low patients included *ECHDC3*, *CHRNA2*, *ALOX15B*, *HMGCS2*, *HSD17B6*, *MSMB*, *POTEB2*, *POTEE*, *OLFM4*, and *POTEB*, which have collectively been implicated in metabolic regulation, androgen metabolism, luminal differentiation, and tumor suppression.^35–39^ Higher expression of these genes in PSA-Low patients indicates retained luminal differentiation and intact androgen-regulated transcriptional programs. Conversely, *TBX1*, a T-box transcription factor involved in developmental signaling, *SURF2*, a component of the surfeit gene locus implicated in ribosomal biogenesis, and *ATRX*, a chromatin remodeling gene associated with genomic instability, were expressed at higher levels in PSA-High patients, potentially reflecting a less differentiated or more treatment-refractory tumor biology.^40^^,^^41^ AR signaling activity and NEPC signature scores were similar between groups at baseline; however, these findings warrant further investigation given the complexity of AR signaling, intratumoral heterogeneity, and the potential for dynamic changes during therapy that may not be captured by pretreatment tumor signatures.

No significant differences in immune cell infiltration were observed between PSA response groups. M2 macrophages and neutrophils were the predominant immune cell populations in both groups, consistent with the immunosuppressive tumor microenvironment characteristic of prostate cancer. The lack of differential immune infiltration suggests that baseline immune composition may not predict depth of PSA response. However, these findings are based on pretreatment samples and do not capture treatment-induced changes in the tumor immune microenvironment that may contribute to outcomes.

Patients achieving deep PSA response had significantly improved OS, which persisted on multivariable analysis. These findings are consistent with data from randomized controlled trials demonstrating the prognostic significance of PSA response for mAPMS. Early PSA response may serve as a clinical tool to identify patients who may benefit from further treatment intensification or deintensification or alternative strategies. The ongoing response-adaptive trials TRIPLE-SWITCH (SWOG/CCTG-PR26; NCT06592924) and A-DREAM (Alliance A032101; NCT05241860), which prospectively assign treatment based on depth of PSA response, provide a unique opportunity to validate and extend these molecular findings.

Several limitations should be considered when interpreting these results. First, this was a retrospective analysis of a real-world database. Second, the PSA threshold of 0.1 ng/mL was selected to provide a more stringent definition of deep PSA response compared to higher cutpoints (0.2 or 4 ng/mL), but alternative thresholds may yield different results. Third, tumor samples were collected at baseline prior to treatment initiation, and molecular profiles may change under the selective pressure of therapy. Fourth, while our multivariable models adjusted for key factors, unmeasured covariates, specifically performance status and disease volume, remain a limitation and potential source of residual confounding. Fifth, OS data is relatively immature. Given censoring is heavily clustered early in the follow-up period, the median OS estimate for the PSA-High group is reached late when the number of patients at risk is low. Thus, while the absolute median survival time warrants cautious interpretation, the overall HR serves as the more robust comparative metric. Overall, the final sample size was relatively small, and these findings require validation in larger cohorts.

In conclusion, these hypothesis-generating findings provide molecular insights into the biological correlates of PSA response in mAPMS and highlight the potential utility of early PSA response as a prognostic marker. Validation in independent prospective cohorts will be essential to determine whether baseline molecular features can inform treatment intensification or de-escalation strategies in mAPMS.

## Supplementary Material

oyag368_Supplementary_Data

## Data Availability

The data that support the findings of this study were derived from the Tempus Lens de-identified multimodal database and are not publicly available due to data use agreements and patient privacy considerations. De-identified data may be made available from Tempus AI, Inc. upon reasonable request and with appropriate institutional and contractual approvals. Requests for access should be directed to the corresponding author, who will facilitate communication with Tempus AI, Inc.
