# Supplementary material for: Genomic and transcriptomic correlates of deep prostate–specific antigen response in patients with metastatic androgen pathway modulation–sensitive prostate cancer
Source: Oncologist. 2026 Sep 17;31(11):oyag368. doi: 10.1093/oncolo/oyag368 (PMC13653217; doi:10.1093/oncolo/oyag368)
Supplement: Supplementary file 1 [file oyag368_supplementary_data.zip › PSA_SupplementaryMaterials_08052026_TABLES.docx]

**Supplementary Table 1.** Baseline patients and disease characteristics by PSA response group. Deep PSA Response defined as a >95% decline from baseline PSA at 6 months; No Deep PSA Response defined as a ≤95% decline.

| **Characteristic** | **No Deep PSA Response**  **Median (Q1, Q3) or N (%)**  **(n=55)** | **Deep PSA Response**  **Median (Q1, Q3) or N (%)**  **(N=366)** | **P-Value** |
| --- | --- | --- | --- |
| **Age at Diagnosis** | 70 (60, 72) | 67 (61, 73) | 0.6 |
|  |  |  |  |
| **Race** |  |  | **0.045** |
| White | 39 (71%) | 196 (54%) |  |
| Asian | 1 (1.8%) | 7 (1.9%) |  |
| Black/African American | 2 (3.6%) | 61 (17%) |  |
| Other Race | 3 (5.5%) | 30 (8.2%) |  |
| Unknown | 10 (18%) | 72 (20%) |  |
|  |  |  |  |
| **Ethnicity** |  |  | 0.8 |
| Not Hispanic | 37 (67%) | 225 (61%) |  |
| Hispanic | 2 (3.6%) | 20 (5.5%) |  |
| Unknown | 16 (29%) | 121 (33%) |  |
|  |  |  |  |
| **Time from Documented Primary Diagnosis to Initial Metastatic Diagnosis** |  |  | **0.032** |
| Within 3 months | 42 (84%) | 326 (93%) |  |
| 3 to 6 months | 0 (0%) | 5 (1.4%) |  |
| More than 6 months | 8 (16%) | 19 (5.4%) |  |
| Unknown | 5 | 16 |  |
|  |  |  |  |
| **Sites of Metastatic Disease** |  |  |  |
| Bone | 27 (49%) | 250 (68%) | **0.005** |
| Lymph node | 16 (29%) | 130 (36%) | 0.3 |
| Lung | 1 (1.8%) | 18 (4.9%) | 0.5 |
| Liver | 0 (0%) | 8 (2.2%) | 0.6 |
|  |  |  |  |
| **Baseline PSA, ng/mL*** | 1 (0, 5) | 42 (11, 141) | **<0.001** |
|  |  |  |  |
| **Time from Metastatic Diagnosis to First-Line Systemic Therapy for mAPMS, months** | 1.15 (0.56, 2.89) | 0.72 (0.21, 1.53) | **0.002** |
|  |  |  |  |
| **Time from Sample Collection to First-Line Systemic Therapy for mAPMS, months** | -0.95 (-2.63, -0.46) | -0.62 (-1.41, -0.20) | **0.008** |
|  |  |  |  |
| **First-Line Systemic Therapy for mAPMS** |  |  | 0.05 |
| ADT Monotherapy | 13 (24%) | 58 (16%) |  |
| ADT + ARPI | 33 (60%) | 233 (64%) |  |
| ADT + ARPI + Docetaxel | 8 (15%) | 65 (18%) |  |
| ADT + Docetaxel | 1 (1.8%) | 10 (2.7%) |  |

*Within 45 days of first line treatment start.

**Abbreviations:** ADT, androgen deprivation therapy; ARPI, androgen receptor pathway inhibitor; mAPMS, metastatic androgen pathway modulation–sensitive prostate cancer; PSA, prostate-specific antigen; Q1, first quartile; Q3, third quartile.

**Note:** P-values were calculated using Wilcoxon rank-sum test for continuous variables and Fisher's exact test or Pearson's chi-squared test for categorical variables.

**Supplementary Table 2.** Unadjusted and adjusted associations between genomic alterations and deep PSA response (PSA <0.1 ng/mL at 6 months).

|  | **Unadjusted Analysis** | | | | **Adjusted Analysis** | | | |
| --- | --- | --- | --- | --- | --- | --- | --- | --- |
| **Gene** | **PSA-High n (%) N=285** | **PSA-Low n (%) N=240** | **OR (95% CI)** | **p-value*** | **PSA-High n (%) N=237** | **PSA-Low n (%) N=183** | **Adjusted OR**  **(95% CI)** | **p-value** |
| ***SPOP*** | 30 (10.5%) | 40 (16.7%) | 1.70  (1.02-2.83) | **0.039** | 27 (11.4%) | 28 (15.3%) | 1.41  (0.79-2.50) | 0.24 |
| ***ZFHX3*** | 17 (6.0%) | 6 (2.5%) | 0.40  (0.16-1.04) | **0.053** | 12 (5.1%) | 5 (2.7%) | 0.54  (0.18-1.57) | 0.26 |
| ***RB1*** | 9 (3.2%) | 15 (6.2%) | 2.04  (0.88-4.76) | 0.091 | 6 (2.5%) | 12 (6.6%) | 2.44  (0.88-6.76) | 0.086 |
| ***JAK1*** | 13 (4.6%) | 5 (2.1%) | 0.45  (0.16-1.27) | 0.12 | 13 (5.5%) | 3 (1.6%) | Not estimable | — |
| ***ZMYM3*** | 13 (4.6%) | 5 (2.1%) | 0.45  (0.16-1.27) | 0.12 | 12 (5.1%) | 4 (2.2%) | Not estimable | — |
| ***APC*** | 27 (9.5%) | 33 (13.8%) | 1.52  (0.89-2.62) | 0.12 | 25 (10.5%) | 27 (14.8%) | 1.66  (0.91-3.02) | 0.096 |
| ***KMT2D*** | 11 (3.9%) | 16 (6.7%) | 1.78  (0.81-3.91) | 0.15 | 10 (4.2%) | 13 (7.1%) | 1.87  (0.79-4.43) | 0.16 |
| ***FOXA1*** | 41 (14.4%) | 25 (10.4%) | 0.69  (0.41-1.18) | 0.2 | 33 (13.9%) | 18 (9.8%) | 0.70  (0.38-1.29) | 0.25 |
| ***BRCA2*** | 18 (6.3%) | 9 (3.8%) | 0.58  (0.25-1.31) | 0.2 | 15 (6.3%) | 7 (3.8%) | 0.66  (0.26-1.67) | 0.38 |
| ***CTNNB1*** | 19 (6.7%) | 10 (4.2%) | 0.61  (0.28-1.34) | 0.2 | 16 (6.8%) | 10 (5.5%) | 0.81  (0.35-1.87) | 0.62 |
| ***PTEN*** | 71 (24.9%) | 49 (20.4%) | 0.77  (0.51-1.17) | 0.2 | 56 (23.6%) | 38 (20.8%) | 0.81  (0.50-1.31) | 0.4 |
| ***CKS1B*** | 11 (3.9%) | 5 (2.1%) | 0.53  (0.18-1.55) | 0.2 | 9 (3.8%) | 4 (2.2%) | Not estimable | — |
| ***FOXO3*** | 5 (1.8%) | 8 (3.3%) | 1.93  (0.62-5.98) | 0.2 | 4 (1.7%) | 6 (3.3%) | Not estimable | — |
| ***ATM*** | 8 (2.8%) | 11 (4.6%) | 1.66  (0.66-4.20) | 0.3 | 7 (3.0%) | 7 (3.8%) | 1.23  (0.42-3.59) | 0.71 |
| ***MAP3K1*** | 10 (3.5%) | 13 (5.4%) | 1.57  (0.68-3.66) | 0.3 | 10 (4.2%) | 11 (6.0%) | 1.71  (0.69-4.24) | 0.24 |
| ***LRP1B*** | 32 (11.2%) | 32 (13.3%) | 1.22  (0.72-2.05) | 0.5 | 27 (11.4%) | 21 (11.5%) | 1.02  (0.55-1.89) | >0.9 |
| ***HDAC2*** | 9 (3.2%) | 10 (4.2%) | 1.33  (0.53-3.34) | 0.5 | 9 (3.8%) | 8 (4.4%) | 1.08  (0.41-2.89) | 0.87 |
| ***GATA1*** | 7 (2.5%) | 8 (3.3%) | 1.37  (0.49-3.83) | 0.5 | 7 (3.0%) | 6 (3.3%) | 1.16  (0.37-3.58) | 0.8 |
| ***PIK3R1*** | 7 (2.5%) | 8 (3.3%) | 1.37  (0.49-3.83) | 0.5 | 5 (2.1%) | 7 (3.8%) | 2.14  (0.65-7.03) | 0.21 |
| ***GRIN2A*** | 16 (5.6%) | 11 (4.6%) | 0.81  (0.37-1.78) | 0.6 | 13 (5.5%) | 7 (3.8%) | 0.72  (0.28-1.85) | 0.49 |
| ***PTPRD*** | 21 (7.4%) | 15 (6.2%) | 0.84  (0.42-1.66) | 0.6 | 17 (7.2%) | 9 (4.9%) | 0.70  (0.30-1.63) | 0.41 |
| ***APLNR*** | 23 (8.1%) | 17 (7.1%) | 0.87  (0.45-1.67) | 0.7 | 21 (8.9%) | 14 (7.7%) | 0.89  (0.43-1.82) | 0.75 |
| ***FAS*** | 11 (3.9%) | 11 (4.6%) | 1.20  (0.51-2.81) | 0.7 | 9 (3.8%) | 10 (5.5%) | 1.46  (0.57-3.74) | 0.43 |
| ***TP53*** | 89 (31.2%) | 71 (29.6%) | 0.93  (0.64-1.34) | 0.7 | 76 (32.1%) | 55 (30.1%) | 0.93  (0.61-1.42) | 0.74 |
| ***BCL11B*** | 10 (3.5%) | 7 (2.9%) | 0.83  (0.31-2.20) | 0.7 | 6 (2.5%) | 6 (3.3%) | 1.30  (0.41-4.16) | 0.66 |
| ***KMT2C*** | 15 (5.3%) | 11 (4.6%) | 0.86  (0.39-1.92) | 0.7 | 15 (6.3%) | 9 (4.9%) | 0.75  (0.31-1.78) | 0.51 |
| ***ERCC3*** | 17 (6.0%) | 16 (6.7%) | 1.13  (0.56-2.28) | 0.7 | 15 (6.3%) | 11 (6.0%) | 1.01  (0.45-2.27) | >0.9 |
| ***KDM6A*** | 14 (4.9%) | 11 (4.6%) | 0.93  (0.41-2.09) | 0.9 | 11 (4.6%) | 9 (4.9%) | 1.02  (0.41-2.55) | >0.9 |
| ***PTPRT*** | 10 (3.5%) | 9 (3.8%) | 1.07  (0.43-2.68) | 0.9 | 7 (3.0%) | 6 (3.3%) | 1.04  (0.34-3.19) | 0.94 |
| ***CDKN1B*** | 9 (3.2%) | 8 (3.3%) | 1.06  (0.40-2.78) | >0.9 | 9 (3.8%) | 8 (4.4%) | 1.28  (0.47-3.48) | 0.63 |
| ***ASXL1*** | 9 (3.2%) | 2 (0.8%) | Not estimable | 0.064 | 9 (3.8%) | 2 (1.1%) | Not estimable | — |
| ***AKT1*** | 10 (3.5%) | 3 (1.3%) | Not estimable | 0.1 | 9 (3.8%) | 3 (1.6%) | Not estimable | — |
| ***NOTCH3*** | 4 (1.4%) | 8 (3.3%) | Not estimable | 0.14 | 3 (1.3%) | 5 (2.7%) | Not estimable | — |

*Pearson’s Chi-squared test.

**Abbreviations:** CI, confidence interval; OR, odds ratio; PSA, prostate-specific antigen.

**Supplementary Table 3.** Unadjusted and adjusted associations between genomic alterations and deep relative PSA response (>95% decline from baseline PSA at 6 months).

|  | **Unadjusted Analysis** | | | | **Adjusted Analysis** | | | |
| --- | --- | --- | --- | --- | --- | --- | --- | --- |
| **Gene** | **No Deep Response n (%) N=55** | **Deep Response (>95%) n (%) N=366** | **OR (95% CI)** | **p-value*** | **No Deep Response n (%) N=55** | **Deep Response (>95%) n (%) N=365** | **Adjusted OR (95% CI)** | **p-value** |
| ***JAK1*** | 7 (12.7%) | 9 (2.5%) | 0.17 (0.06-0.49) | **0.002** | 7 (12.7%) | 9 (2.5%) | 0.24 (0.06-0.94) | **<0.05** |
| ***ZMYM3*** | 5 (9.1%) | 11 (3.0%) | 0.31 (0.10-0.93) | **0.045** | 5 (9.1%) | 11 (3.0%) | 0.10 (0.02-0.59) | **<0.05** |
| ***TP53*** | 24 (43.6%) | 108 (29.5%) | 0.54 (0.30-0.96) | **0.035** | 24 (43.6%) | 107 (29.3%) | 0.63 (0.31-1.26) | 0.191 |
| ***PTEN*** | 17 (30.9%) | 77 (21.0%) | 0.60 (0.32-1.11) | 0.10 | 17 (30.9%) | 77 (21.1%) | 0.91 (0.43-1.92) | 0.812 |
| ***KMT2C*** | 5 (9.1%) | 19 (5.2%) | 0.55 (0.20-1.53) | 0.2 | 5 (9.1%) | 19 (5.2%) | 0.87 (0.25-3.04) | 0.832 |
| ***PTPRD*** | 5 (9.1%) | 21 (5.7%) | 0.61 (0.22-1.69) | 0.4 | 5 (9.1%) | 21 (5.8%) | 0.53 (0.15-1.82) | 0.312 |
| ***APC*** | 9 (16.4%) | 44 (12.0%) | 0.70 (0.32-1.52) | 0.4 | 9 (16.4%) | 43 (11.8%) | 0.53 (0.19-1.46) | 0.216 |
| ***APLNR*** | 6 (10.9%) | 29 (7.9%) | 0.70 (0.28-1.78) | 0.4 | 6 (10.9%) | 29 (7.9%) | 0.70 (0.23-2.11) | 0.522 |
| ***FOXA1*** | 8 (14.5%) | 43 (11.7%) | 0.78 (0.35-1.77) | 0.6 | 8 (14.5%) | 43 (11.8%) | 0.74 (0.26-2.11) | 0.578 |
| ***LRP1B*** | 5 (9.1%) | 43 (11.7%) | 1.33 (0.50-3.52) | 0.6 | 5 (9.1%) | 43 (11.8%) | 1.03 (0.33-3.21) | 0.965 |
| ***SPOP*** | 6 (10.9%) | 49 (13.4%) | 1.26 (0.51-3.10) | 0.6 | 6 (10.9%) | 49 (13.4%) | 0.47 (0.13-1.67) | 0.244 |
| ***FLCN*** | 4 (7.3%) | 3 (0.8%) | Not estimable | 0.007 | 4 (7.3%) | 3 (0.8%) | Not estimable | — |
| ***DNM2*** | 3 (5.5%) | 1 (0.3%) | Not estimable | 0.008 | 3 (5.5%) | 1 (0.3%) | Not estimable | — |
| ***MUTYH*** | 2 (3.6%) | 0 (0.0%) | Not estimable | 0.017 | 2 (3.6%) | 0 (0.0%) | Not estimable | — |
| ***MSH2*** | 3 (5.5%) | 3 (0.8%) | Not estimable | 0.032 | 3 (5.5%) | 3 (0.8%) | Not estimable | — |
| ***NOTCH3*** | 3 (5.5%) | 5 (1.4%) | Not estimable | 0.073 | 3 (5.5%) | 5 (1.4%) | Not estimable | — |
| ***SMAD2*** | 2 (3.6%) | 2 (0.5%) | Not estimable | 0.085 | 2 (3.6%) | 2 (0.5%) | Not estimable | — |
| ***PPM1D*** | 2 (3.6%) | 2 (0.5%) | Not estimable | 0.085 | 2 (3.6%) | 2 (0.5%) | Not estimable | — |
| ***HNF1A*** | 2 (3.6%) | 4 (1.1%) | Not estimable | 0.2 | 2 (3.6%) | 4 (1.1%) | Not estimable | — |
| ***BCL11B*** | 3 (5.5%) | 9 (2.5%) | Not estimable | 0.2 | 3 (5.5%) | 9 (2.5%) | Not estimable | — |
| ***PIK3CA*** | 3 (5.5%) | 9 (2.5%) | Not estimable | 0.2 | 3 (5.5%) | 9 (2.5%) | Not estimable | — |
| ***CTNNB1*** | 1 (1.8%) | 25 (6.8%) | Not estimable | 0.2 | 1 (1.8%) | 25 (6.8%) | Not estimable | — |
| ***WT1*** | 2 (3.6%) | 5 (1.4%) | Not estimable | 0.2 | 2 (3.6%) | 5 (1.4%) | Not estimable | — |
| ***MSH6*** | 2 (3.6%) | 5 (1.4%) | Not estimable | 0.2 | 2 (3.6%) | 5 (1.4%) | Not estimable | — |
| ***FAS*** | 4 (7.3%) | 15 (4.1%) | Not estimable | 0.3 | 4 (7.3%) | 15 (4.1%) | Not estimable | — |
| ***PPP2R2A*** | 2 (3.6%) | 7 (1.9%) | Not estimable | 0.3 | 2 (3.6%) | 7 (1.9%) | Not estimable | — |
| ***BRCA2*** | 1 (1.8%) | 21 (5.7%) | Not estimable | 0.3 | 1 (1.8%) | 21 (5.7%) | Not estimable | — |
| ***CKS1B*** | 3 (5.5%) | 10 (2.7%) | Not estimable | 0.4 | 3 (5.5%) | 10 (2.7%) | Not estimable | — |
| ***RNF43*** | 2 (3.6%) | 8 (2.2%) | Not estimable | 0.6 | 2 (3.6%) | 8 (2.2%) | Not estimable | — |
| ***MSH3*** | 2 (3.6%) | 9 (2.5%) | Not estimable | 0.6 | 2 (3.6%) | 9 (2.5%) | Not estimable | — |
| ***MS4A1*** | 2 (3.6%) | 9 (2.5%) | Not estimable | 0.6 | 2 (3.6%) | 9 (2.5%) | Not estimable | — |
| ***IKZF1*** | 2 (3.6%) | 10 (2.7%) | Not estimable | 0.7 | 2 (3.6%) | 10 (2.7%) | Not estimable | — |
| ***PIK3R1*** | 2 (3.6%) | 10 (2.7%) | Not estimable | 0.7 | 2 (3.6%) | 10 (2.7%) | Not estimable | — |
| ***AKT1*** | 2 (3.6%) | 10 (2.7%) | Not estimable | 0.7 | 2 (3.6%) | 10 (2.7%) | Not estimable | — |
| ***PTPRT*** | 2 (3.6%) | 11 (3.0%) | Not estimable | 0.7 | 2 (3.6%) | 11 (3.0%) | Not estimable | — |
| ***ERCC3*** | 4 (7.3%) | 22 (6.0%) | Not estimable | 0.8 | 4 (7.3%) | 22 (6.0%) | Not estimable | — |
| ***KMT2D*** | 3 (5.5%) | 20 (5.5%) | Not estimable | >0.9 | 3 (5.5%) | 20 (5.5%) | Not estimable | — |
| ***MAP3K1*** | 2 (3.6%) | 19 (5.2%) | Not estimable | >0.9 | 2 (3.6%) | 19 (5.2%) | Not estimable | — |
| ***GRIN2A*** | 2 (3.6%) | 18 (4.9%) | Not estimable | >0.9 | 2 (3.6%) | 18 (4.9%) | Not estimable | — |
| ***KDM6A*** | 2 (3.6%) | 18 (4.9%) | Not estimable | >0.9 | 2 (3.6%) | 18 (4.9%) | Not estimable | — |
| ***RB1*** | 2 (3.6%) | 16 (4.4%) | Not estimable | >0.9 | 2 (3.6%) | 16 (4.4%) | Not estimable | — |
| ***ZFHX3*** | 2 (3.6%) | 15 (4.1%) | Not estimable | >0.9 | 2 (3.6%) | 15 (4.1%) | Not estimable | — |
| ***CDKN1B*** | 2 (3.6%) | 15 (4.1%) | Not estimable | >0.9 | 2 (3.6%) | 15 (4.1%) | Not estimable | — |
| ***HDAC2*** | 2 (3.6%) | 15 (4.1%) | Not estimable | >0.9 | 2 (3.6%) | 15 (4.1%) | Not estimable | — |
| ***ATM*** | 1 (1.8%) | 13 (3.6%) | Not estimable | >0.9 | 1 (1.8%) | 13 (3.6%) | Not estimable | — |
| ***GATA1*** | 1 (1.8%) | 12 (3.3%) | Not estimable | >0.9 | 1 (1.8%) | 12 (3.3%) | Not estimable | — |
| ***CDK12*** | 1 (1.8%) | 11 (3.0%) | Not estimable | >0.9 | 1 (1.8%) | 11 (3.0%) | Not estimable | — |

*Pearson’s Chi-squared test.

**Abbreviations:** CI, confidence interval; OR, odds ratio; PSA, prostate-specific antigen.

**Supplementary Table 4.** Tumor immune cell infiltration by PSA response group. PSA-Low defined as PSA <0.1 ng/mL at 6 months; PSA-High defined as PSA ≥0.1 ng/mL at 6 months.

| **Cell Type** | **PSA-High (N=214)** | **PSA-Low (N=177)** | **p-value** |
| --- | --- | --- | --- |
| M2 Macrophages | 0.06 (0.05, 0.07) | 0.06 (0.05, 0.08) | 0.2 |
| CD8+ T Cells | 0.00 (0.00, 0.01) | 0.00 (0.00, 0.01) | 0.3 |
| CD4+ T Cells | 0.00 (0.00, 0.00) | 0.00 (0.00, 0.00) | 0.3 |
| Dendritic Cells | 0.00 (0.00, 0.00) | 0.00 (0.00, 0.00) | 0.4 |
| Neutrophils | 0.06 (0.05, 0.10) | 0.07 (0.05, 0.10) | 0.4 |
| Regulatory T Cells | 0.02 (0.01, 0.04) | 0.02 (0.01, 0.03) | 0.4 |
| Total CD4+ | 0.02 (0.01, 0.04) | 0.02 (0.01, 0.04) | 0.4 |
| M1 Macrophages | 0.01 (0.00, 0.02) | 0.01 (0.00, 0.02) | 0.6 |
| NK Cells | 3.53 (2.90, 4.29) | 3.65 (2.94, 4.28) | 0.7 |
| B Cells | 0.04 (0.03, 0.05) | 0.04 (0.03, 0.05) | 0.7 |
| Monocytes | 0.00 (0.00, 0.00) | 0.00 (0.00, 0.00) | 0.9 |

**Abbreviations:** NK, natural killer; PSA, prostate-specific antigen.

**Note:** Values represent estimated cell fractions derived from RNA sequencing data using the quanTIseq algorithm. Data presented as median (Q1, Q3). P-values were calculated using Wilcoxon rank-sum test.
