# Supplementary material for: Genomic and transcriptomic correlates of deep prostate–specific antigen response in patients with metastatic androgen pathway modulation–sensitive prostate cancer
Source: Oncologist. 2026 Sep 17;31(11):oyag368. doi: 10.1093/oncolo/oyag368 (PMC13653217; doi:10.1093/oncolo/oyag368)
Supplement: Supplementary file 1 [file oyag368_supplementary_data.zip › PSA_SupplementaryMaterials_08052026_FIGURES.docx]

**Supplementary Figure 1.** Consort Flow Diagram. Patients were identified from the Tempus Lens database with a primary diagnosis of prostate cancer, xT (DNA; 648 genes) sequencing, and curated treatment information (n=11,534). Of these, 8,001 patients had a metastatic prostate cancer diagnosis within 90 days of sample collection. Patients were then filtered to include those who received a specified first-line treatment regimen [androgen deprivation therapy (ADT) alone, ADT plus androgen receptor pathway inhibitor (ARPI), ADT plus docetaxel, or ADT plus ARPI plus docetaxel), yielding 5,126 patients. After excluding 102 patients with curated castration resistant prostate cancer notation prior to or within 90 days of ADT start, 5,024 patients remained. Samples were classified as non-castrate (n=4,324) or castrate (n=790), with 90 patients contributing samples to both groups (total samples: 5,114; unique patients: 5,024). Among non-castrate patients, 4,285 had specimens collected within 12 months prior to or within 90 days of first-line treatment start. Of these, 618 patients had prostate specific antigen (PSA) data available, and 543 had PSA values at 6 months. The final cohort consisted of 525 patients with non-castrate metastatic prostate cancer who had both PSA data and qualifying biopsies.

**Abbreviations:** ADT, androgen deprivation therapy; ARPI, androgen receptor pathway inhibitor; CRPC, castration-resistant prostate cancer; Doce, docetaxel; Dx, diagnosis; LoT, line of therapy; mPCa, metastatic prostate cancer; PSA, prostate-specific antigen; Tx, treatment; xT, Tempus xT next-generation sequencing assay.

**Supplementary Figure 2.** A) Oncoplot among PSA-high group. B) Oncoplot among PSA-low group. PSA-Low was defined as PSA <0.1 ng/mL and PSA-High as PSA ≥0.1 ng/mL at 6 months after initiation of first-line systemic therapy.

A)

B)
